# Segregation by Payer in Obstetrics and Gynecology Residency Ambulatory Care Sites

**DOI:** 10.1001/jamanetworkopen.2024.34347

**Published:** 2024-09-18

**Authors:** Kavita Vinekar, Neena Qasba, Hannah Reiser, Erika Banks, Kavita S. Arora, Brownsyne Tucker Edmonds, Karen George

**Affiliations:** 1 Department of Obstetrics and Gynecology, Sidney Kimmel Medical College of Thomas Jefferson University, Philadelphia, Pennsylvania; 2 Department of Obstetrics and Gynecology, University of Connecticut School of Medicine, Farmington; 3 Department of Obstetrics and Gynecology, David Geffen School of Medicine, University of California, Los Angeles; 4 Department of Obstetrics and Gynecology, New York University Grossman Long Island School of Medicine, Mineola; 5 Department of Obstetrics and Gynecology, University of North Carolina at Chapel Hill, Chapel Hill; 6 Department of Obstetrics and Gynecology, Indiana University School of Medicine, Indianapolis; 7 Department of Obstetrics, Gynecology, and Reproductive Science, Larner College of Medicine, University of Vermont, Burlington

## Abstract

**Importance:**

Many teaching hospitals in the US segregate patients by insurance status, with resident clinics primarily composed of publicly insured or uninsured patients and faculty practices seeing privately insured patients. The prevalence of this model in obstetrics and gynecology residencies is unknown.

**Objectives:**

To examine the prevalence of payer-based segregation in obstetrics and gynecology residency ambulatory care sites nationally and to compare residents’ and program directors’ perceptions of differences in quality of care between payer-segregated and integrated sites.

**Design, Setting, and Participants:**

This national survey study included all 6060 obstetrics and gynecology residents and 293 obstetrics and gynecology residency program directors in the US as of January 2023. The proportion of program directors reporting payer segregation was calculated to characterize the national prevalence of this model in obstetrics and gynecology. Perceived differences in care quality were compared between residents and program directors at payer-segregated sites.

**Main Outcome and Measures:**

The primary measure was prevalence of payer-based segregation in obstetrics and gynecology residency programs in the US as reported by residency program directors. The secondary measure was resident and program director perceptions of care quality in these ambulatory care settings. Before study initiation, the study hypothesis was that residents and program directors at ambulatory sites with payer-based segregation would report more disparity in perceived health care quality between resident and faculty practices compared with those from integrated sites.

**Results:**

A total of 251 residency program directors (response rate, 85.7%) and 3471 residents (response rate, 57.3%) were included in the study. Resident respondent demographics reflected demographics of obstetrics and gynecology residents nationally in terms of racial and ethnic distribution (6 [0.2%] American Indian or Alaska Native; 425 [13.0%] Asian; 239 [7.3%] Black or African American; 290 [8.9%] Hispanic, Latinx, or Spanish; 7 [0.2%] Native Hawaiian or Other Pacific Islander; 2052 [62.7%] non-Hispanic White; 49 [1.5%] multiracial; 56 [1.7%] other [any race not listed]; and 137 [4.2%] preferred not to say) and geographic distribution (regional prevalence of payer-based segregation: 36 of 53 [67.9%] in the Northeast, 35 of 44 [79.5%] in the Midwest, 43 of 67 [64.2%] in the South, and 13 of 22 [59.1%] in the West), with 2837 respondents (86.9%) identifying as female. Among program directors, 127 (68.3%) reported payer-based segregation in ambulatory care. University programs were more likely to report payer-based segregation compared with community, hybrid, and military programs (63 of 85 [74.1%] vs 31 of 46 [67.4%], 32 of 51 [62.7%], and 0, respectively; *P* = .04). Residents at payer-segregated programs were less likely than their counterparts at integrated programs to report equal or higher care quality from residents compared with faculty (1662 [68.7%] vs 692 [81.6%] at segregated and integrated programs, respectively; *P* < .001).

**Conclusions and Relevance:**

In this survey study of residents and residency program directors, payer-based segregation was prevalent in obstetrics and gynecology residency programs, particularly at university programs. These findings reveal an opportunity for structural reform to promote more equitable care in residency training programs.

## Key Points

**Question:**

What is the prevalence of payer-based segregation (ie, separation of faculty vs resident-led care based on a patient’s insurance status) in obstetrics and gynecology residency ambulatory settings?

**Findings:**

In this national survey study of 251 obstetrics and gynecology residency program directors and 3471 residents, the prevalence of payer-based segregation was 68% overall and 74% at university-based programs.

**Meaning:**

Payer-based segregation was prevalent in obstetrics and gynecology residency programs, particularly at university programs, revealing an opportunity for a national movement toward integration in medical training.

## Teaser

This survey study assesses whether residents and program directors at ambulatory sites with payer-based segregation report more disparity in perceived health care quality between resident and faculty practices compared with those from integrated sites.

## Introduction

The separation of resident physician and faculty physician ambulatory care in US teaching hospitals is a long-standing tradition, characterized by the disproportionate funneling of publicly insured and uninsured patients to resident clinics and privately insured patients to faculty practices.^1^ In the US, where more than half of Medicaid enrollees belong to ethnic or racial minority groups,^2^ this payer-based segregation often translates to racial segregation.^3^

Structural racism has led to staggering inequities in reproductive health outcomes nationally, with Black pregnant individuals being 3 times more likely to die in childbirth compared with their White counterparts.^4^ In the context of larger national movements toward antiracism to address these inequities, segregation in health care, and specifically in medical education, is a potential focus area for institutional reform.^1,5,6,7,8,9,10^ Although previous studies^3,11^ have investigated the payer and racial separation present in residency programs, the national prevalence of this payer-based segregation of resident and faculty care has not been previously studied. Moreover, the prevalence of this payer-based segregation in obstetrics and gynecology ambulatory care has not previously been described.

We sought to characterize the prevalence of payer-based segregation in obstetrics and gynecology residency programs in the US and to compare resident and program director perceptions of care quality in these ambulatory care settings. We hypothesized that residents and program directors at ambulatory sites with payer-based segregation would report more disparity in perceived health care quality between resident and faculty practices compared with those from integrated sites.

## Methods

We conducted a national survey study of all US obstetrics and gynecology residents (N = 6060) and program directors (N = 293) in January 2023 using 2 electronic surveys administered at the time of annual in-service examination by the Council on Resident Education in Obstetrics and Gynecology. Both groups were informed regarding the survey purpose and provided written informed consent to participate at the time of the survey. Responses were anonymous and not linked to individual residency programs. Respondents who did not consent to the survey or left the entirety of the survey blank were excluded from the analysis. For the resident survey, respondents from international training programs (n = 115) and those who were not resident physicians (n = 17) were excluded. This study was deemed exempt by the institutional review board of Health Media Lab because it carried no more than minimal risk to respondents because the responses were anonymously collected. The study followed the American Association for Public Opinion Research (AAPOR) reporting guideline.^12^

The survey contained demographic questions, including self-reported race and ethnicity. This information was included because understanding the experiences of physicians of varied racial and ethnic identities could inform future efforts toward inclusion and equity. In addition to demographic information, each survey included Likert scale questions about ambulatory care sites, perceived demographic differences in patient populations, and perceived differences in care quality (eFigure in Supplement 1). The questions were developed and piloted by several of the authors across institutions and edited for clarity and applicability to a range of program settings. The author group responded to a request for proposals of survey questions and were selected by the Council on Resident Education in Obstetrics and Gynecology executive leadership team for inclusion in the annual resident and program director surveys.

Respondent residence by US geographic region was also reported. The categories were as follows: (1) the Northeast, which included New England (Maine, New Hampshire, Vermont, Massachusetts, Rhode Island, and Connecticut) and the Middle Atlantic region (New Jersey, New York, and Pennsylvania); (2) the Midwest, which included the East North Central (Ohio, Michigan, Indiana, Illinois, and Wisconsin) and West North Central (Minnesota, Kansas, Nebraska, Iowa, Montana, North Dakota, and South Dakota) regions; (3) the South, which included the West South Central (Arkansas, Louisiana, Oklahoma, and Texas), East South Central (Alabama, Kentucky, Mississippi, and Tennessee), and South Atlantic (Delaware, Washington, DC, Florida, Georgia, Maryland, North Carolina, South Carolina, Virginia, and West Virginia) regions; (4) the West, which included the Mountain (Arizona, Colorado, Idaho, Montana, Nevada, New Mexico, Utah, and Wyoming) and Pacific (Alaska, California, Hawaii, Oregon, and Washington) regions; and (5) Puerto Rico.

Sites were considered to have payer-based ambulatory care segregation if respondents stated that residents were “much more likely” or “more likely” to see publicly insured patients (Medicaid or Medicare) compared with faculty practices. Because 1 program director was surveyed per residency program, the program director survey was used for determining national prevalence of payer-based segregation in ambulatory care at obstetrics and gynecology training programs.

### Statistical Analysis

In addition to payer-based segregation, we investigated spatial separation of resident and faculty practices. Respondents were considered to have spatially separated ambulatory care sites if they reported that the primary resident continuity ambulatory site was spatially separated from faculty practices within the same building or in a different building from faculty practices. We performed descriptive analysis using χ^2^ testing in Stata SE, version 16 (StataCorp LLC), with an a priori determination of a 2-sided *P* < .05 for statistical significance.

## Results

**Figure 1.**
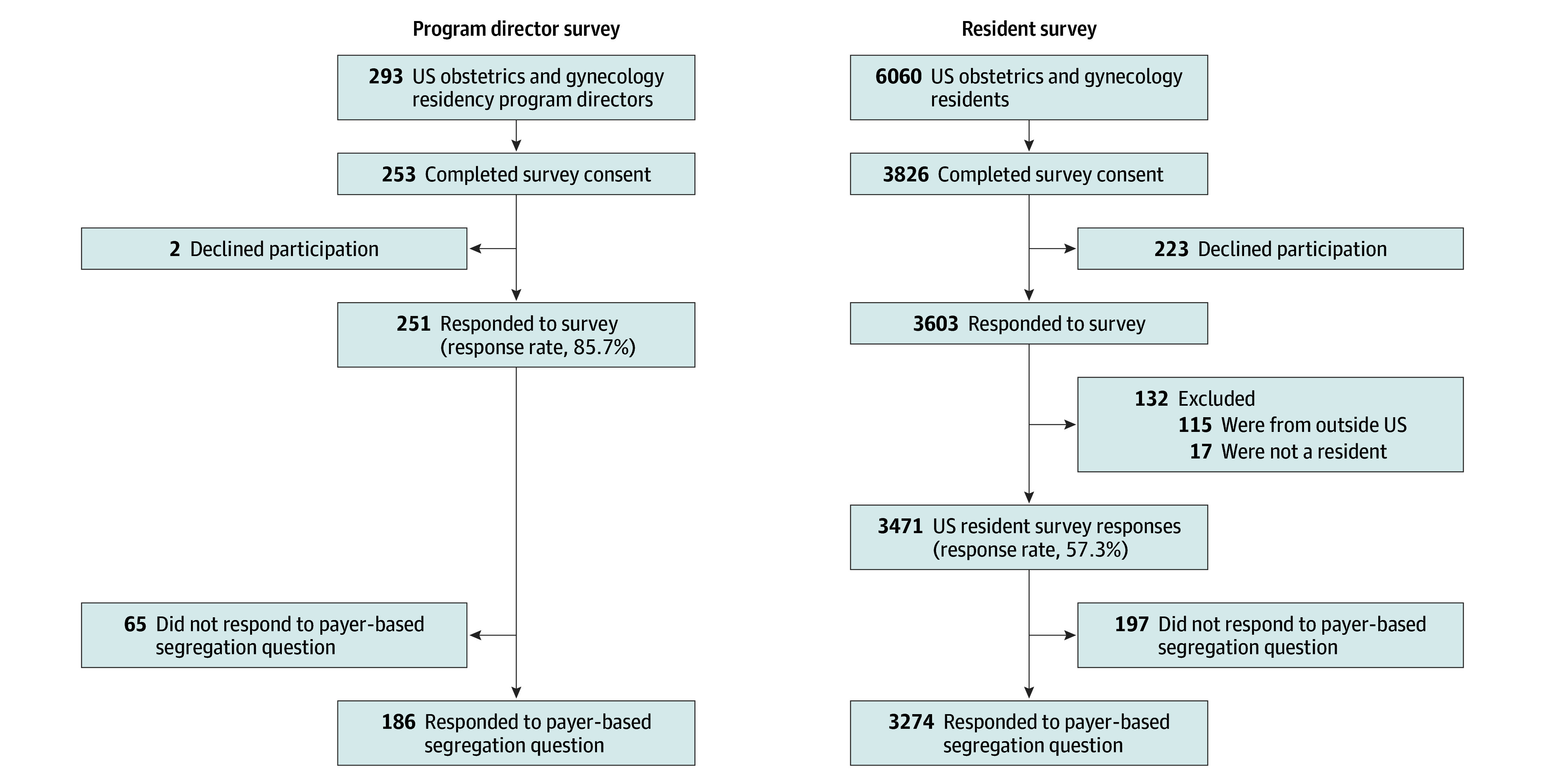
Obstetrics and Gynecology Program Director and Resident Survey Response Rates, January 2023 National In-Service Examination Survey

A total of 251 residency program directors (response rate, 85.7%) and 3471 residents (response rate, 57.3%) were studied. Of the respondents, 186 program directors (74.1%) and 3274 residents (94.3%) answered the question about payer-based segregation (Figure 1). Overall, resident respondent demographics aligned with reported demographics of obstetrics and gynecology residents nationally^13^: 2837 (86.9%) identified as female, 379 (11.6%) as male, 11 (0.3%) as nonbinary, 1 (0.03%) gender not listed, and 38 (1.2%) preferred not to answer; 1386 (42.4%) were from university-based programs, with 847 (25.9%) and 1000 (30.6%) from community and community-university hybrid programs, respectively. Overall, 6 resident respondents (0.2%) identified as American Indian or Alaska Native; 425 (13.0%) as Asian; 239 (7.3%) as Black or African American; 290 (8.9%) as Hispanic, Latinx, or Spanish; 7 (0.2%) as Native Hawaiian or Other Pacific Islander; 2052 (62.7%) as non-Hispanic White; 49 (1.5%) as multiracial; 56 (1.7%) as other (any race not listed); and 137 (4.2%) preferred not to say (Table 1). Residents at payer-segregated sites differed from those at integrated sites by program size, program type, geographic region, and race and ethnicity at payer-segregated vs payer-integrated programs.

**Table 1.** Resident Respondent Demographics by Payer-Based Ambulatory Site Segregation^a^

Demographic	Residents			*P* value^b^
	From all programs (N = 3274)	At payer-segregated programs (n = 2423)	At payer-integrated programs (n = 851)	
Postgraduate year				
1	816 (24.9)	565 (23.3)	251 (29.5)	.003
2	845 (25.8)	631 (26.0)	214 (25.2)	
3	842 (25.7)	633 (26.1)	209 (24.6)	
4	771 (23.6)	594 (24.6)	177 (20.8)	
No. of residents per year				
≤4	950 (29.2)	664 (69.9)	286 (33.7)	<.001
5-7	1456 (44.7)	1035 (43.0)	421 (49.7)	
8-9	505 (15.5)	426 (17.7)	79 (9.3)	
≥10	344 (10.6)	282 (11.7)	62 (7.3)	
Program type				
University based	1386 (42.4)	1028 (42.6)	358 (42.1)	<.001
Community based	847 (25.9)	604 (25.0)	243 (28.6)	
Combination community and university based	1000 (30.6)	779 (32.2)	221 (26.0)	
Military based	33 (1.0)	5 (0.2)	28 (3.3)	
Geographic region				
Northeast	926 (28.3)	685 (28.3)	241 (28.3)	<.001
Midwest	835 (25.5)	673 (27.8)	162 (19.0)	
South	1059 (32.4)	801 (33.1)	258 (30.3)	
West	426 (13.0)	246 (10.2)	180 (21.2)	
Puerto Rico	28 (0.9)	18 (0.7)	10 (1.2)	
Race and ethnicity				
American Indian or Alaska Native	6 (0.2)	4 (0.2)	2 (0.2)	<.001
Asian	426 (13.0)	302 (12.5)	124 (14.6)	
Black or African American	239 (7.3)	168 (6.9)	71 (8.3)	
Hispanic, Latinx, or Spanish	290 (8.9)	198 (8.2)	92 (10.8)	
Native Hawaiian or Other Pacific Islander	7 (0.2)	3 (0.1)	4 (0.5)	
Non-Hispanic White	2052 (62.7)	1583 (65.3)	469 (55.1)	
Multiracial	49 (1.5)	31 (1.3)	18 (2.1)	
Other race not listed	56 (1.7)	38 (1.6)	18 (2.1)	
Prefer not to say	137 (4.2)	90 (3.7)	47 (5.5)	
Gender identity				
Female	2837 (86.9)	2111 (87.3)	726 (85.5)	.22
Male	379 (11.6)	268 (11.1)	111 (13.1)	
Nonbinary	11 (0.3)	8 (0.3)	3 (0.9)	
Not listed	1 (0.03)	0	1 (0.1)	
Prefer not to answer	38 (1.2)	30 (1.2)	3 (0.4)	
Resident and faculty ambulatory sites				
Shared space with faculty	1764 (54.1)	1097 (45.4)	667 (78.9)	<.001
Spatially separated, same building as faculty	548 (16.8)	457 (18.9)	91 (10.8)	
Different building from faculty	839 (25.7)	792 (32.8)	47 (5.6)	
Another type of building	25 (0.8)	21 (0.9)	4 (0.5)	
Unsure	70 (2.2)	45 (1.9)	25 (3.0)	
Prefer not to answer	15 (0.5)	4 (0.2)	11 (1.3)	
Perceived resident care quality compared with faculty care				
Higher quality	303 (9.3)	250 (10.3)	53 (6.3)	<.001
Equal quality	2051 (62.8)	1412 (58.4)	639 (75.4)	
Lesser quality	446 (13.7)	421 (17.4)	25 (3.0)	
Do not know	424 (13.0)	315 (13.0)	109 (12.9)	
Prefer not to answer	43 (1.3)	21 (0.9)	22 (2.6)	
Perceived difference in race and ethnicity of patients in resident vs attending practices	1249 (38.2)	1176 (48.7)	73 (8.6)	<.001

^a^ Data are presented as No. (%).

^b^ A χ^2^ test was used to compare resident responses at payer-segregated vs payer-integrated programs. A 2-sided *P* < .05 was considered significant.

We found that 127 program directors (68.3%) reported payer-based segregation. University programs were more likely to report payer-based segregation compared with community, hybrid, and military programs (63 of 85 [74.1%] vs 31 of 46 [67.4%], 32 of 51 [62.7%], and 0, respectively; *P* = .04). A total of 82 program directors (44.1%) reported spatial segregation between resident and faculty practices, with 61 (32.8%) reporting resident ambulatory care in a different building from faculty practices (Table 2). The geographic composition of payer-segregated vs payer-integrated sites differed. The regional prevalence of payer-based segregation was 36 of 53 (67.9%) in the Northeast, 35 of 44 (79.5%) in the Midwest, 43 of 67 (64.2%) in the South, and 13 of 22 (59.1%) in the West.

**Table 2.** Program Director Report of Program Demographics by Payer-Based Ambulatory Site Segregation Status^a^

Demographic	Programs			*P* value^b^
	All programs (N = 186)	Payer segregated (n = 127)	Payer integrated (n = 59)	
No. of residents per year				
≤4	63 (34.2)	38 (30.2)	25 (43.1)	.19
5-7	82 (44.6)	60 (47.6)	22 (37.9)	
8-9	21 (11.4)	17 (13.5)	4 (6.9)	
≥10	18 (9.8)	11 (8.7)	7 (12.1)	
Program type				
University based	85 (45.6)	63 (50.0)	22 (37.3)	.04
Community based	46 (24.9)	31 (24.6)	15 (25.4)	
Combination community and university based	51 (27.6)	32 (25.4)	19 (32.2)	
Military based	3 (1.6)	0 (0)	3 (5.1)	
Geographic region				
Northeast	53 (28.5)	36 (28.4)	17 (28.8)	.27
Midwest	44 (23.7)	35 (27.6)	9 (15.3)	
South	67 (36.0)	43 (33.9)	24 (40.7)	
West	22 (11.8)	13 (10.2)	9 (15.3)	
Resident ambulatory clinic location				
Main hospital	71 (38.2)	48 (37.8)	23 (39.0)	.65
Offsite hospital-owned clinic	75 (40.3)	53 (41.7)	22 (37.3)	
Physician-owned group practice	23 (12.4)	17 (13.4)	6 (10.2)	
Federally qualified health center	10 (5.4)	5 (3.9)	5 (8.5)	
Other	7 (3.8)	4 (3.2)	3 (5.1)	
Resident and faculty ambulatory sites				
Shared space with faculty	100 (53.8)	49 (38.6)	51 (86.4)	<.001
Spatially separated, same building as faculty	21 (11.3)	19 (15.0)	2 (3.4)	
Different building from faculty	61 (32.8)	56 (44.1)	5 (8.5)	
Other	4 (2.2)	3 (2.4)	1 (1.7)	
Perceived resident care quality compared to faculty care				
Higher quality	24 (12.9)	19 (15.0)	5 (8.5)	.10
Equal quality	137 (73.7)	88 (69.3)	49 (83.1)	
Lesser quality	18 (9.7)	16 (12.6)	2 (3.4)	
Do not know	7 (3.8)	4 (3.2)	3 (5.1)	
Equal quality of care (resident vs faculty)	137 (73.7)	88 (69.3)	49 (83.1)	.047
Lesser quality of care (resident vs faculty)	18 (9.7)	16 (12.6)	2 (3.4)	.048
Higher quality of care (resident vs faculty)	24 (12.9)	19 (15.0)	5 (8.5)	.22

^a^ Data are presented as No. (%).

^b^ A χ^2^ test was used to compare resident responses at payer-segregated vs payer-integrated programs. A 2-sided *P* < .05 was considered significant.

**Figure 2.**
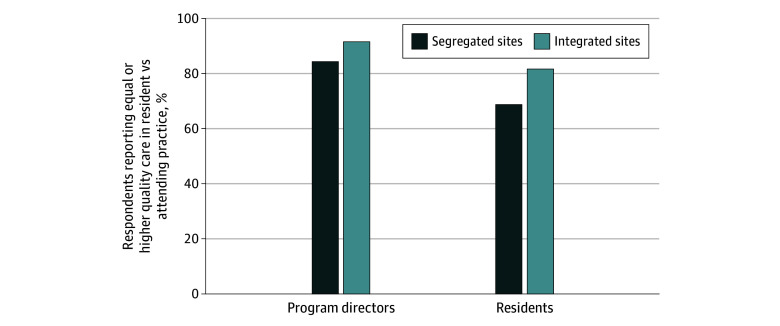
Perceived Differences in Care Quality in Resident and Faculty Practices at Payer-Segregated vs Payer-Integrated Ambulatory Care Sites, Comparing Obstetrics and Gynecology Resident and Program Director Perceptions A χ^2^ test was used to compare resident responses at payer-segregated vs payer-integrated programs. For payer-segregated sites, *P* < .001. For payer-integrated sites, *P* = .03.

Residents at payer-segregated programs were more likely than those at integrated programs to report perceived racial and ethnic differences between patients seen at resident vs faculty practices (1176 of 2423 [48.5%] vs 73 of 851 [8.6%] at segregated vs integrated programs, respectively; *P* < .001). They were also more likely than those at integrated programs to report unequal care quality between resident and faculty care (Table 2). This trend was noted at 2 extremes: in payer-segregated programs, residents were more likely to report lower quality of care delivery with residents than with faculty (421 of 2423 [17.4%] vs 25 of 851 [2.9%] at segregated vs integrated programs, respectively). However, residents at segregated programs were also more likely to report that residents delivered higher quality care than faculty (250 of 2423 [10.3%] vs 53 of 851 [6.2%] at segregated and integrated programs, respectively*)*. Residents at payer-segregated programs were less likely to report equal or higher care quality from residents compared with faculty (1662 [68.7%] vs 692 [81.6%] at segregated and integrated programs, respectively; *P* < .001) (Figure 2). Program directors were less likely than residents to report quality disparities between resident and faculty care (Figure 2).

## Discussion

We found that more than two-thirds of obstetrics and gynecology residency programs and nearly three-quarters of university-based residency programs reported payer-based segregation of resident and faculty practices. Spatial separation of these practices was also prevalent at more than half of all programs. Although payer segregation was prevalent at most sites in all regions, the Midwest was notable for the highest prevalence of this structuring. Of note, residents were more likely to report perceived differences in care quality at payer-segregated sites compared with integrated sites. Residents were more likely than program directors to report differences in care quality between resident and faculty care.

Our findings add to a limited body of literature on this subject, including a survey of primary care sites demonstrating that higher Medicaid enrollment was associated with the presence of resident practitioners.^3^ Notably, in this study, we explored perceived differences in care quality rather than specific measured outcomes. There is an increasing body of evidence comparing resident and attending outcomes with objective health care measures. Several of these studies^14,15,16,17,18^ have shown poorer care delivery, patient care use, and patient satisfaction in resident compared with faculty practices. Others have demonstrated poor continuity of care in resident ambulatory care settings.^19^ In contrast, others have shown similar or improved objective health measures and rates of patient satisfaction in resident ambulatory care settings.^20,21,22^ Of note, these studies^20,21,22^ compared resident-led care to attending-led care but did not compare payer-segregated to payer-integrated ambulatory care settings. In our study, we compared perceptions of care quality (by residents and program directors) because this may capture nuances of care quality that may not be easily reflected in objective measures.

Interestingly, we found that program directors and residents had differing perceptions of care quality between residents and faculty based on whether patients were segregated by insurance type. Although most residents overall reported equal care quality between residents and faculty, residents were more likely than program directors to report disparate care quality between resident and faculty care. Of note, residents had disagreement in their reports of disparate care quality, with some residents reporting higher care quality in resident practices compared with faculty practices and others reporting lower care quality. This finding may reflect heterogeneity in many factors, such as time allotted for visits, local or institutional resources (eg, social work services and care coordinators), access measures (eg, appointment wait times), continuity of care, and variation in supervision in the resident practice setting.

In the US, where racial and ethnic minority individuals are more likely to have public health insurance, payer-based inequities disproportionately impact minoritized communities.^2,3^ In our study, half of residents from payer-segregated programs reported racial and ethnic differences in patient populations seen by residents compared with faculty. These structural inequities necessitate reform, particularly in obstetrics and gynecology, with mortality in Black pregnant individuals exceeding that of their White counterparts by 3-fold.^23^ Efforts to understand and address racial segregation in health care may be instrumental in addressing racial health inequities. The racial implications of payer-based segregation may also have potential legal ramifications.

Payer-based segregation may negatively impact learners and their future medical practice. One survey of third-year medical students revealed that payer-based segregation was commonly observed across medical specialties, incurring emotional exhaustion and moral injury among trainees.^11^ A previous study^24^ in internal medicine, although not focused on payer-based segregation specifically, found that dysfunctional and underresourced outpatient ambulatory care settings have worsened resident burnout and affected resident decision-making about subspecialty selection.^24^ Although our study did not specifically explore the association of payer-based segregation with learner morale, the association of this separation with resident experiences and future practice is an area for further study. Moreover, the long-term implications of segregation in residency ambulatory care, particularly the concern for inequitable care provision by graduated residents in clinical practice based on patients’ insurance status,^25^ remains an area of future investigation.

As institutions identify their own structures of segregation within training programs, it is important to seek models of integration to guide restructuring efforts. At the University of North Carolina at Chapel Hill, for example, integration efforts included a baseline internal review detailing the disparate demographics of resident clinic patients compared with faculty practice, demonstrating that patients in the resident practice were more likely to be uninsured or publicly insured, were more likely to travel greater distances, and had longer appointment wait times. Resident and faculty practices were subsequently integrated, with schedulers blinded to patient demographics and insurance status, to ensure that clinical acuity and patient preferences were prioritized. Patient access metrics continue to be tracked to ensure equitable care for all patients.^5^ For other residency programs planning to restructure, seeking models such as the University of North Carolina at Chapel Hill may provide important guidance for integration efforts.

This study is the first, to our knowledge, to characterize the national prevalence of payer-segregated ambulatory care in US residency training programs and specifically in obstetrics and gynecology programs. Further research is needed to characterize the prevalence of this segregation in other medical fields, understand associations with patient experiences and health outcomes, explore the effects of this structuring on trainees, and inform efforts at institutional reformation toward more equitable models of care.

### Strengths and Limitations 

A strength of our study is that the data were obtained from 2 large national surveys of all obstetrics and gynecology program directors and residents with high response rates. Additionally, we characterized the prevalence of payer-based segregation in obstetrics and gynecology residency training programs, which had not previously been explored.

Our study also has some limitations. The brief survey used likely does not capture nuances about programmatic structuring, referral and scheduling practices, or local resources. We could not capture individual definitions of care quality, and comparative assessments of quality are subjective. Future studies may compare objective health outcome measures as a reflection of care quality. Our study is also limited by the potential for nonresponse bias, especially in the resident survey, for which the response rate was lower. However, the similarities in demographic composition between respondents and obstetrics and gynecology residents nationally suggest that respondents were generally representative of the total surveyed population. Because the survey was administered immediately after the annual resident in-service examination, it is possible that respondents’ stress levels may have influenced their responses. Furthermore, it is notable that one-quarter of responding program directors did not respond to the question about payer-based segregation. It is possible that social desirability bias may have influenced responses (or nonresponse) to these questions. If this is true, our reported prevalence of payer segregation may be an underestimate.

Of note, although each responding program produced 1 program director response, allowing for prevalence measurement, the resident responses were deidentified and not linked to specific programs. Thus, comparison of resident and program director survey responses, particularly regarding perceived care quality, may have been limited by an inability to adjust for clustering of residents within individual programs.

We used the term public insurance, thereby grouping patients with Medicaid and Medicare, recognizing that most publicly insured patients seen in obstetrics and gynecology resident-led ambulatory settings have Medicaid and relatively few patients have Medicare.^26^ Future work, particularly in gynecologic subspecialties or in other medical specialties, may provide further insight into organizational structures for older patients and those with disabilities and severe health conditions qualifying for Medicare.

## Conclusions

Payer-based segregation in obstetrics and gynecology residency ambulatory care settings exists in most programs, with higher rates at university-based programs. This segregation is associated with greater perceived inequities in health care delivery for patients seeing residents vs faculty. These findings reveal an opportunity for reform in medical training programs to promote more equitable health care for patients and more just models of health care delivery for trainees.

## Notes

Supplement 1. **eFigure.** Ambulatory Care Structure Survey Questions Administered at the Time of Annual In-Service Examination by the Council on Resident Education in Obstetrics and Gynecology, January 2023

Supplement 2.
Data Sharing Statement
