# Supplementary material for: Segregation by Payer in Obstetrics and Gynecology Residency Ambulatory Care Sites
Source: JAMA Netw Open. 2024 Sep 18;7(9):e2434347. doi: 10.1001/jamanetworkopen.2024.34347 (PMC11411379; doi:10.1001/jamanetworkopen.2024.34347)
Supplement: Supplement 1. — eFigure. Ambulatory Care Structure Survey Questions Administered at the Time of Annual In-Service Examination by the Council on Resident Education in Obstetrics and Gynecology, January 2023 [file jamanetwopen-e2434347-s001.pdf]

Supplementary Online Content

Vinekar K, Qasba N, Reiser H, et al. Segregation by payer in obstetrics and gynecology residency ambulatory care sites. *JAMA Netw Open*. 2024;7(9):e2434347. doi:10.1001/jamanetworkopen.2024.34347

eFigure. Ambulatory Care Structure Survey Questions Administered at the Time of Annual In-Service Examination by the Council on Resident Education in Obstetrics and Gynecology, January 2023

This supplementary material has been provided by the authors to give readers additional information about their work.

© 2024 Vinekar K et al. *JAMA Network Open.*

eFigure. Ambulatory Care Structure Survey Questions Administered at the Time of Annual In-Service Examination by the Council on Resident Education in Obstetrics and Gynecology, January 2023

The ambulatory site where the majority of faculty see patients is located:
(a) in the main hospital
(b) in an offsite hospital-owned clinic
(c) in a physician-owned group practice with hospital affiliation
(d) in a federally qualified health center
(e) other
(f) I don't know
(g) prefer not to answer

The primary resident continuity ambulatory site is located:
(a) in a shared space with attending/faculty clinics
(b) spatially separated from attending/faculty clinics, within the same building
(c) in a different building from attending/faculty clinics
(d) other
(e) I don't know
(f) prefer not to answer

*In my opinion, residents see patients of similar racial/ethnic backgrounds when compared with attending/faculty practices.**

Strongly agree/Agree/Somewhat agree/Somewhat disagree/Disagree/Strongly disagree/ N/A / I don't know / Prefer not to answer

From my perspective, resident continuity ambulatory care sites are ________ to be publicly insured (Medicaid, Medicare) or uninsured when compared to patients in attending/faculty practices.

Much more likely/more likely/equally likely/less likely/much less likely/ N/A / I don't know / Prefer not to answer

In my opinion, patients seen in the resident ambulatory sites receive ______ quality care compared to the patients in attending/faculty practices.

(a) higher
(b) equal
(c) lesser
(d) I don't know
(e) prefer not to answer

*this question appeared only in the resident survey

© 2024 Vinekar K et al. *JAMA Network Open.*
