# Supplementary material for: Segregation by Payer in Obstetrics and Gynecology Residency Ambulatory Care Sites
Source: JAMA Netw Open. 2024 Sep 18;7(9):e2434347. doi: 10.1001/jamanetworkopen.2024.34347 (PMC11411379; doi:10.1001/jamanetworkopen.2024.34347)
Supplement: Supplement 2. — Data Sharing Statement [file jamanetwopen-e2434347-s002.pdf]

Data Sharing Statement

Vinekar. Segregation by Payer in Obstetrics and Gynecology Residency Ambulatory Care Sites. *JAMA Netw Open*. Published September 18, 2024.
doi:10.1001/jamanetworkopen.2024.34347

Data
Data available: No

Additional Information
Explanation for why data not available: Confidential survey data cannot be publicly shared, but deidentified data can be shared individually upon request
